# Molecular and biological characterization of an Asian-American isolate of Chikungunya virus

**DOI:** 10.1371/journal.pone.0266450

**Published:** 2022-04-06

**Authors:** Edwin D. Archila, Lady S. López, Jaime E. Castellanos, Eliana P. Calvo

**Affiliations:** Laboratorio de Virología, Universidad El Bosque, Bogotá D.C., Colombia; CEA, FRANCE

## Abstract

Chikungunya virus is an arthropod-transmitted virus that causes chikungunya fever, a disease characterized by severe muscle and joint pain. In 2013, the virus was introduced to the Americas and caused approximately 2.7 million cases of infection during the subsequent two years. The lack of knowledge regarding the biological behavior of the viral strains circulating during the outbreak motivated the characterization of an isolate from the Colombian outbreak, starting from analysis of the complete genome to the biological behavior in vitro. The full genome was retrieved using next-generation sequencing. The infective and replicative capacities were evaluated in HEK293T, Huh-7, and MRC-5 cell lines. The infection rates were determined by flow cytometry, and the cytopathic effect was assessed by a resazurin fluorescent metabolic assay. The viral yield was quantified using the virus plaque formation assay, while the viral proteins and genomic RNA kinetics were subsequently evaluated by western-blot and RT-qPCR. The COL7624 isolate clustered with other American and Caribbean sequences in the Asian American lineage. The T669A substitution in E2 protein distinguished it from other Colombian sequences reported in 2014. After 48 h post infection (hpi), the three cell lines analyzed reached infection percentages exceeding 65%, generating a high load of infectious viral progeny. The infection kinetics indicated that the replication peak of this CHIKV isolate is around 24 hpi, although gRNA is detectable in the culture supernatant from 4 hpi onwards. The infection caused the overexpression of interferon and pro-inflammatory cytokines, such as IL-1β, TNF-α, and IL-8. The COL7624 CHIKV isolate exhibited a high infective and replicative capacity as well as activation of cellular immune responses, similar to isolates belonging to the other genotypes.

## Introduction

Chikungunya fever is a viral infection that is caused by the chikungunya virus (CHIKV). The virus was first detected in 1952 in Tanzania and it was restricted to sporadic outbreaks in other countries, mainly across the African and Asian continents [1]. The first major epidemic of Chikungunya fever was documented on the island of La Réunion between 2005 and 2006, affecting more than one-third of its population [2], and during 2007–2009 the virus spread to European countries for the first time [3].

Between 2011 and 2013, the disease affected several countries in Central and West Africa, Southeast Asia, and the South Pacific region [4–8]. At the end of 2013, the first cases were documented in Saint Martin Island and then spread throughout the Americas, affecting over 2.7 million people in 44 territories. Approximately 80% of the cases during the epidemic were concentrated in Brazil, the Dominican Republic, Colombia, Costa Rica, and Guadeloupe [9]. After the large epidemic in the Americas, outbreaks were reported in Italy, India, Pakistan, Bangladesh, and Kenya during 2016–2017, and in Thailand during 2018–2019 [10–13].

CHIKV is an arbovirus transmitted by mosquitoes *Aedes aegypti* and *Ae albopictus*, belongs to the Togaviridae family and the *Alphavirus* genus. It is a member of the old world arthritogenic alphaviruses, which cause febrile illness characterized by arthralgias and myalgias of varying intensities [2]. The viral genome is a positive-sense, single-stranded RNA of approximately 11.8 kb with a methyl guanosine cap at the 5’ end and a poly adenine tail at the 3’ end. It encodes for two open reading frames (ORFs) separated by a short, non-coding 76 nt region. The first ORF encodes the four non-structural proteins (nsP1–nsP4), while the second ORF encodes three structural proteins (capsid, E1, and E2) and two small peptides (E3 and 6K) [14]. Additionally, the 5’ and 3’ non-translated regions and nsP1 contain conserved sequence elements (ESCs) that give rise to structural elements essential for replication and genome packaging [15].

So far, three CHIKV genotypes have been defined, i.e., the Asian, West African (WA), and East, Central, and Southern African (ECSA), along with two lineages, i.e., the Indian Ocean Lineage (IOL) and the Asian/American Lineage (AAL), which were derived from the ECSA and Asian genotypes, respectively [16, 17]. The IOL was responsible for the 2004 and 2009 outbreaks in La Réunion, Southeast Asia, India, Italy, and France [8, 18–20]. It is characterized by an alanine to valine substitution at position 226 of the envelope glycoprotein E1 (A226V), which has been attributed to the adaptation of the virus to the *Ae albopictus* mosquito [21]. The AAL was responsible for the 2013–2016 outbreaks in the American continents. It is defined by two substitutions at E2 (V368A) and 6K (L20M), and duplication of 177 nucleotides at the 3’ untranslated region (3’UTR) end that confers increased replication in mosquito cells [7, 16, 22, 23].

In the Americas, the disease affected individuals of all ages and socioeconomic strata, with clinical manifestations ranging from mild to severe, along with atypical manifestations such as encephalitis, meningoencephalitis, myocarditis, nephritis, pulmonary edema, muco-cutaneous lesions, purpuric lesions, necrosis, sepsis, and septic shock, among others [24–28]. Although only 440 deaths were reported [9], an increase in mortality during the peak of CHIKV circulation was reported in Brazil, Guadeloupe, Martinique, Puerto Rico, and the Dominican Republic [29–32]. In Brazil, a total of 236,287 cases and 120 deaths were reported in 2016; however, Freitas et al. had estimated that 7,231 deaths were associated with the infection [30]. In the Dominican Republic, a similar analysis indicated that 2,853 deaths could have been caused by CHIKV in 2015; although only six were registered to the health system [29].

The rapid spread of the virus, the severity of infection, and the high number of fatal cases associated with CHIKV observed in the Americas and the absence of knowledge regarding the biological behavior of the American isolates motivated the characterization of a Colombian isolate. This study analyzed the complete genome and evaluated the infective and replicative capacities. The activation of the antiviral response in different human cell lines was also investigated.

## Materials and methods

### Virus, cells, and antibodies

CHIKV was isolated from a patient during the 2015 epidemic in Colombia and propagated by four sequential passages in Vero cells. The supernatants were harvested and frozen at -80°C before titration and further use.

The consensus sequence of the whole genome was deposited in the GenBank with the accession number MW656171.1, and accessible at https://www.ncbi.nlm.nih.gov/nuccore/MW656171.1.

Adherent cells such as HEK 293T (ATCC ® CRL-11268G-1™), MRC-5 (ATCC ® CCL-171™), Huh-7 (ATCC® CRL-2117™), BHK (ATCC® CCL-10™), and Vero clone E6 (ATCC® CRL-1586™ cells) were grown in Dulbecco’s modified Eagle’s medium (DMEM) (Millipore-Sigma., MO, USA) supplemented with 10% fetal bovine serum (FBS; Gibco, Thermo Fisher Scientific Corp., CA, USA) at 37°C and 5% CO_2_.

The following antibodies were used in this study: mouse polyclonal antibody directed against E1/E2 protein (anti-E1/E2), which was provided by Anne Claire Brehin from Institut Pasteur, Paris, France; mouse monoclonal anti-Chikungunya virus antibody [3585] (ab155841; Abcam., Cambridge, UK); an in-house obtained rat anti-CHIKV polyclonal serum against recombinant nsP1; an anti-actin mAb (D6A8, Cat. # 8457; Cell Signaling Technology, MA, USA); and anti-Caspase-3 Rabbit mAb (D3R6Y, Cat # 14220; Cell Signaling Technology, MA, USA).

### Cell infection

Human cells were seeded in DMEM and incubated overnight. They were subsequently infected at multiplicity of infection (MOI) values of 0.1, 0.5, and 1. After 1 h of incubation at 37°C, the virus was washed off and DMEM supplemented with 2% FBS was added. After 24 and 48 hours post-infection (hpi), the supernatants were collected for viral titration.

### CHIKV titration

The supernatants from infected cells were titrated following a standard procedure. Briefly, 10-fold dilutions of cell culture supernatants were added into 24-well plates seeded with 100,000 Vero cells and incubated at 37°C under a 5% CO_2_ atmosphere. After 2 h, the virus was removed, and the cell monolayers were overlaid with 1 mL of 2% FBS and 1.5% carboxymethyl cellulose (Millipore-Sigma., MO, USA)/DMEM medium. Three days post-infection, the cell monolayers were fixed and stained with 1% crystal violet. The titer was calculated and presented as the number of plaque-forming units (PFUs) per mL.

### CHIKV detection by flow cytometry

CHIKV-infected and mock-infected cells were cultured for 24 and 48 h, trypsinized, and centrifuged once (900 x g) for 5 min at 4°C with cold phosphate-buffered saline (PBS). The washed cell pellet was resuspended in 100 μL of a Cytofix/Cytoperm solution (BD Pharmingen., CA, USA) and incubated at 4°C for 20 min to improve the permeabilization. The cells were then washed once with perm/wash (BD Pharmingen., CA, USA), and the pellet was resuspended in 50 μL of anti-chikungunya virus antibody (ab155841; Abcam, Cambridge, UK) diluted at a ratio of 1:50 in perm/wash and incubated for 1 h. The cells were again washed with perm/wash, resuspended in 50 μL of labeled anti-mouse IgG-FITC (MilliporeSigma., MO, USA), and incubated at 4°C for 30 min under darkness. The samples were subjected to a washing step, and the final cell pellet was resuspended in 100 μL of PBS and stored at 4°C until acquisition and analysis using a BD Accuri™ C6 Cytometer (BD Biosciences., CA, USA). Acquired data were analyzed using FlowJo software.

### CHIKV detection by immunofluorescence

CHIKV-infected and mock-infected cells were cultured for 24 h and then fixed with methanol: acetone solution (v/v 1:1) for 20 min at 4°C. Fixed cells were permeabilized for 30 min in 0.02% Triton X100 (MilliporeSigma., MO, USA), blocked in 5% goat serum (Vector Laboratories) for 30 min, and then incubated with a 1:50 diluted anti-CHIKV monoclonal antibody (ab155841; Abcam, Cambridge, UK) at 20°C for 1 h. After removing the excess unbound antibodies, goat anti-mouse IgG conjugated to Alexa Fluor 488 (diluted 1:500; Invitrogen, Thermo Fisher Scientific Corp., CA, USA) was added for 30 min at 20°C. The cells were then counterstained with Hoechst stain (Vector Laboratories., CA, USA) and imaged using an Axio Observer microscope (Carl Zeiss AG., Jena, Germany). Images were captured using Axio Vision 4.5 software.

### CHIKV detection by western blot

The cells were lysed in RIPA buffer with a protease inhibitor cocktail (Cell Signaling Technology, Inc., MA, USA) for 30 min at 4°C. Cell lysates obtained by centrifugation for 15 min at 15,000 × g were quantified using the bicinchoninic acid assay. Equal amounts of protein (20 μg) were loaded per lane, resolved on a 10% SDS polyacrylamide gel and, blotted onto a PVDF membrane (Merck Millipore., MA, USA). After blocking with 5% non-fat milk in TBS with 0.1% Tween-20 (TBST), the membranes were incubated for 2 h with rat antisera against CHIKV nsP1 (diluted 1:1,000) or mouse antisera against E1/E2 (diluted 1:1,000) in TBST. A mouse monoclonal antibody against β-actin was used as the loading control. After washing with TBST, anti-mouse or anti-rat IgG conjugated with horseradish peroxidase (Kirkegaard & Perry Lab Inc., MD, USA) was added for 1 h. The bound antibodies were then detected using the West Pico Blotting Detection Reagent (Thermo Fisher Scientific Corp., MA, USA) with a Gel Doc system (Bio-Rad Laboratories, Inc., CA, USA).

### CHIKV RNA detection and quantification by Reverse Transcription-Polymerase Chain Reaction (RT-qPCR)

Before the RNA extraction, RNase A treatment to remove free ssRNA and to assess only genomic RNA to be amplificated was made. Viral RNA was extracted from the cell culture supernatants using the RTP® DNA/RNA Virus Mini Kit (STRATEC SE, Birkenfeld, Germany) following the manufacturer’s instructions. The eluted RNAs were stored at -80°C until use.

The CHIKV genomic copies were determined using the SYBR green-based assay Luna® Universal One-Step RT-qPCR (New England Biolabs., MA, USA) and the E1F: ACGCAATTGAGCGAAGCAC and E1R: CTGAAGACATTGGCCCCAC primers at a concentration of 0.2 μM. All reactions were performed in a 20 μL reaction mix containing 5 μL of the RNA template, with the amplification protocol consisting of an RT step at 55°C for 15 min and RTase inactivation at 95°C for 3 min, followed by 40 cycles of PCR at 95°C for 15 s and 60°C for 45 s. The RNA concentration, expressed as the number of genome copies per mL, was calculated using a standard curve established using known concentrations of a plasmid containing an insert corresponding to the E1 gene.

Total RNA from infected cells was extracted using TRIzol reagent (Invitrogen, Thermo Fisher Scientific Corp., MA, USA). Relative RT-qPCR was conducted using SYBR green-based assay Luna® Universal One-Step RT-qPCR (New England Biolabs., MA, USA) and specific primers for viral RNA (E1, nsP1), along with genes associated with antiviral (IFN-α) and pro-inflammatory responses (IL-1β, IL-6, IFN-γ, and TNF-α). The relative change in expression for each gene was calculated following Pfaffl’s method and represented as the fold change as compared to the mock-infected cells [33].

### Cell viability assay

Briefly, cells were plated in 96-well plates and exposed to the indicated MOI. After 24 and 48 hpi, 10 μL of a 440 mM resazurin solution was added to 90 μL culture medium for 4 h and incubated at 37°C. The fluorescent signal of the resorufin was read using a Tecan Infinite 200 fluorometric reader (Tecan Trading AG, Männedorf, Switzerland) with excitation and emission wavelengths of 530 nm and 590 nm, respectively.

### Genome sequencing and bioinformatic analysis

The sequences generated by Illumina NextSeq500 sequencing were trimmed to remove adaptors from each end using Trimmomatic, and reads shorter than 50 base pairs were discarded. All remaining reads were mapped against the viral reference database using SPAdes [34].

The sequence generated was aligned using the ClustalW function of Bio-Edit 7.2.3 with 82 full-length CHIKV strains representing all major CHIKV genotypes. Owing to the ambiguous alignment of the UTR, only the ORFs comprising 11,256 nucleotides were used to reconstruct the phylogenetic tree and for subsequent analyses. A phylogenetic tree was constructed in MEGA X 10.1.5 following the maximum likelihood method with a bootstrap value of 1,000 replicates [35].

CHIKV isolate H20235 from Saint Martin, collected in 2013 (GenBank ID MG208125.1), was used as the reference strain for the Asian-American genotype. Both the nucleotide and translated amino acid sequences were used for the comparison and mapping of the changes in the Colombian CHIKV isolate.

### Statistical analysis

The experimental data were analyzed by conducting a Kruskal-Wallis test, followed by a Mann-Whitney U test or One Way ANOVA test using GraphPad Prism 7.0a software. The results were presented as the median and interquartile range or mean and standard deviation. Statistical significance was defined as p<0,05.

## Results

### Genomic characterization of the COL7624 CHIKV isolate

The virus used in this study was isolated from the plasma of an individual with febrile syndrome, who had tested positive for CHIKV and negative for dengue virus by RT-qPCR. The sample was collected in Girardot, Colombia in February 2015 and inoculated and passaged four times in Vero cells. The viral harvest was tittered in BHK cells following a plating method, reaching a titer of 3 × 10^7^ PFU/mL. The viral RNA extracted from this isolate named COL7624 was sequenced using Illumina technology, achieving coverage of 99.9%. Of the consensus sequence generated, 11,224 nts corresponded to the coding region, 75 nts to the 5’ UTR end, and 725 to the 3’ UTR. This sequence was 99.9% identical to other Colombian viruses isolated in Santander de Quilichao-Cauca (MH329297.1) and Pereira-Risaralda (MH329298.1) in November 2014 [36], with differences occurring at positions 5382 (nsP3 P1794L) and 10,972 (E2 T669A) on the coding regions. For both untranslated ends, we observed 99% similarity with the Colombian sequence MH329298.1. In the 5’ UTR region, 59 out of the reported 60 nts along with 725 out of the 726 nts in the 3’ UTR were found to be identical.

**Fig 1:**
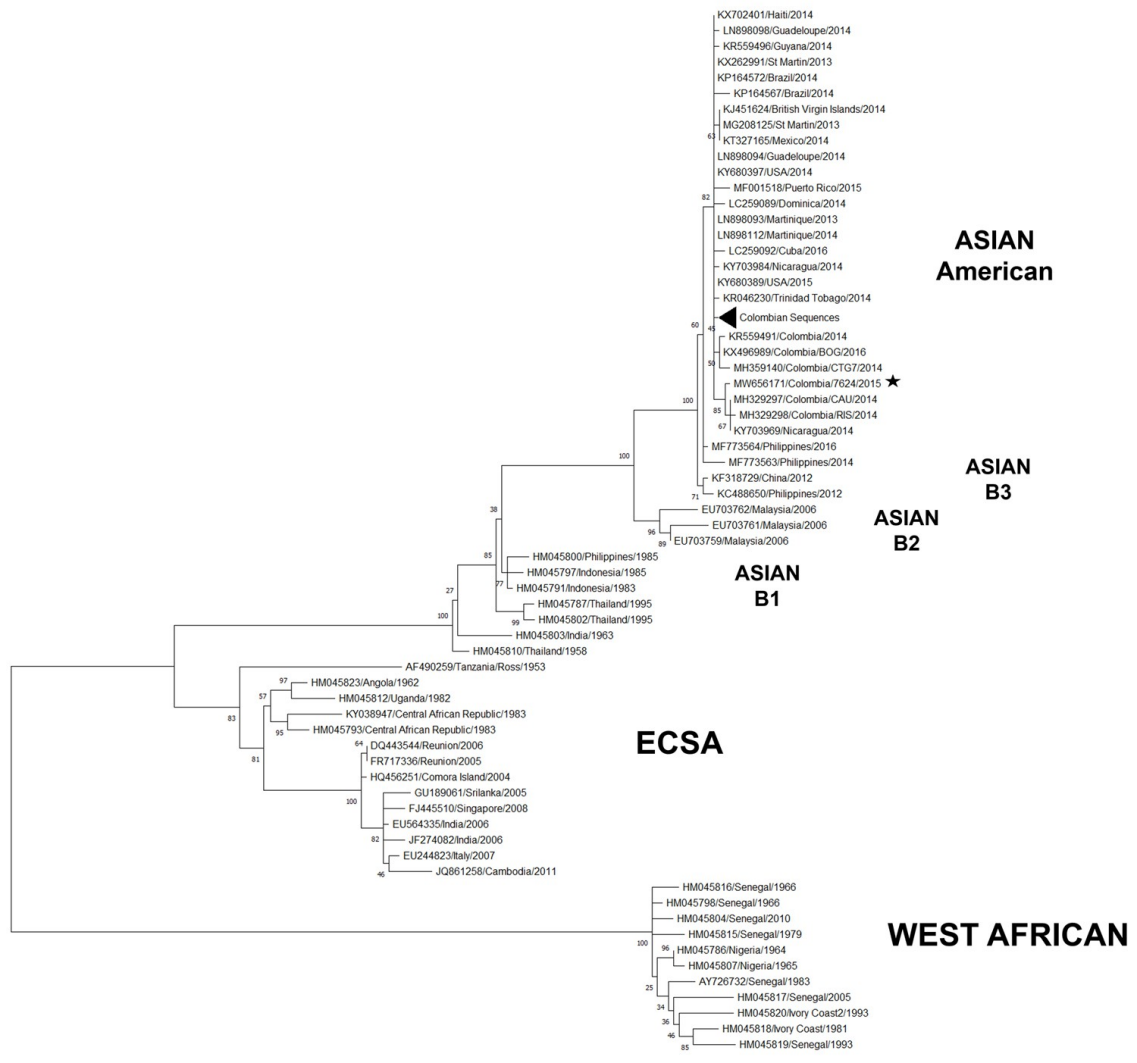
Phylogenetic analysis of Chikungunya virus. Full coding sequences of CHIKV were used. The maximum likelihood tree was constructed using the general time-reversible model and inferred following bootstrap analyses using 1,000 replicates. Strain names are in the format of: accession number/country /year of isolation. CHIKV genotypes: West African, Asian, and East/Central/South African (ECSA) are indicated on the right.

Phylogenetic analysis conducted on the coding region of 82 sequences belonging to the three CHIKV genotypes confirmed that the isolate belonged to the Asian genotype, and clustered with viruses isolated from America and the Caribbean in Asian-American lineage (Fig 1). Interestingly, V>A and L>M substitutions at positions 9,569 (GCG>GTG) and 9,793 (CTG>ATG), and two repeated segments in the 11,672–12,026 region of the 3’UTR were also identified, which are characteristic of this lineage, as reported by Stapleford et al [22].

Sequence analysis indicated that the COL7624 isolate presented three non-synonymous substitutions, with two in nsP2 and one in E2. Moreover, these changes were apparently not the consequence of adaptation to Vero cells, as they have also been observed in viruses obtained directly from patient serum [23] or after a single passage in c6/36 or Vero cells [36] (Table 1).

**Table 1:** Amino acid substitutions distinctive of COL7624 Colombian CHIKV isolate.

Position (nt)	Position (aa)	Gene	Sequences
3232 TAT > CAT	Y 1078 H	nsP2	MH329297.1 Colombia-Cauca (C6/36 / Vero) MH329298.1 Colombia-Risaralda (C6/36 / Vero) KY703969.1 Nicaragua 2014 (not passaged) HM045804.1 Senegal 2010 (C6/36)
3764 GGC > GCC	G 1255 A	nsP2	MH329297.1 Colombia-Cauca MH329298.1 Colombia-Risaralda KY703969.1 Nicaragua
9496 ACA > GCA	T 669 A	E2	HM045821.1 Senegal 1963 AF490259.3 Tanzania 1953 Ross strain

Complete genome analysis of the Asian genotype revealed 28 differences between the isolates collected before 2000 and viruses from more recent outbreaks, which led to the separation into four clades by the presence of specific substitutions. The first clade consisted of viruses recorded before 2000; the second contained sequences from the Malaysia outbreak in 2006, with 18 substitutions compared to the predecessors. The third consisted of sequences obtained in China, the Philippines, and Micronesia between 2010 and 2014 with eight additional mutations. The last one represented viruses isolated from the Americas with the two substitutions typical of the AAL (Fig 1, Table 2).

**Table 2:** Relevant amino acid substitutions identified between Asian genotype clades.

	Position (aa)	Protein	Asian	Asian	Asian	American 2013–17
			<2000	2006	2010–14	B3
			B1	B2	B3	
**Non-structural polyprotein**	121	nsp1	A	A	E	E
	478	nsp1	T	A	A	A
	688	nsp2	V	V	V	V/A*
	808	nsp2	Q	L	L	L
	1303	nsp2	N	S	S	S
	1410	nsp3	S	T	T	T
	1546	nsp3	M	V	V	V
	1557	nsp3	T	T	I	I
	1665	nsp3	Q	R	R	R
	1669	nsp3	T	M	M	M
	1712–15	nsp3	LPTT	LPTI/	**----**	**----**
	1767	nsp3	L	Q	Q	Q
	1770	nsp3	A	A	T	T
	1784–85	nsp3	LQ	LR	FR	FR
	1790–92	nsp3	TTM	ITV	ITV	ITV
	1817	nsp3	E	D	D	D
	2132	nsp4	F	F	L	L
	2134	nsp4	K	R	R	R
**Structural polyprotein**	55	C	A	A	V	V
	280	E3	Q	Q	Q/R	R
	330	E2	N	H	H	H
	531	E2	N	S	S	S
	573	E2	L	S	F	F
	**693**	**E2**	**V**	**V**	**V**	**A**
	696	E2	V	V	L	L
	**768**	**6K**	**L**	**L**	**L**	**M**
	793	6K	T	M	M	M
	1113	E1	P	S	S	S

Codon numbering from the first codon in each open reading frame.

Residues in boldface indicate critical aa changes in Asian American genotype.

*The amino acid substitution was unique to Colombian isolates.

Regarding the substitutions, 18 (64%) were identified in non-structural proteins, and ten in structural proteins. The C-terminal end of nsP3 was the most variable region (Table 2) with the deletion of four amino acids (1712–1715) and six mutations (1716; 1767; 1784–1785; and 1790–1792).

### Phenotypic characterization of the COL7624 CHIKV isolate

**Fig 2:**
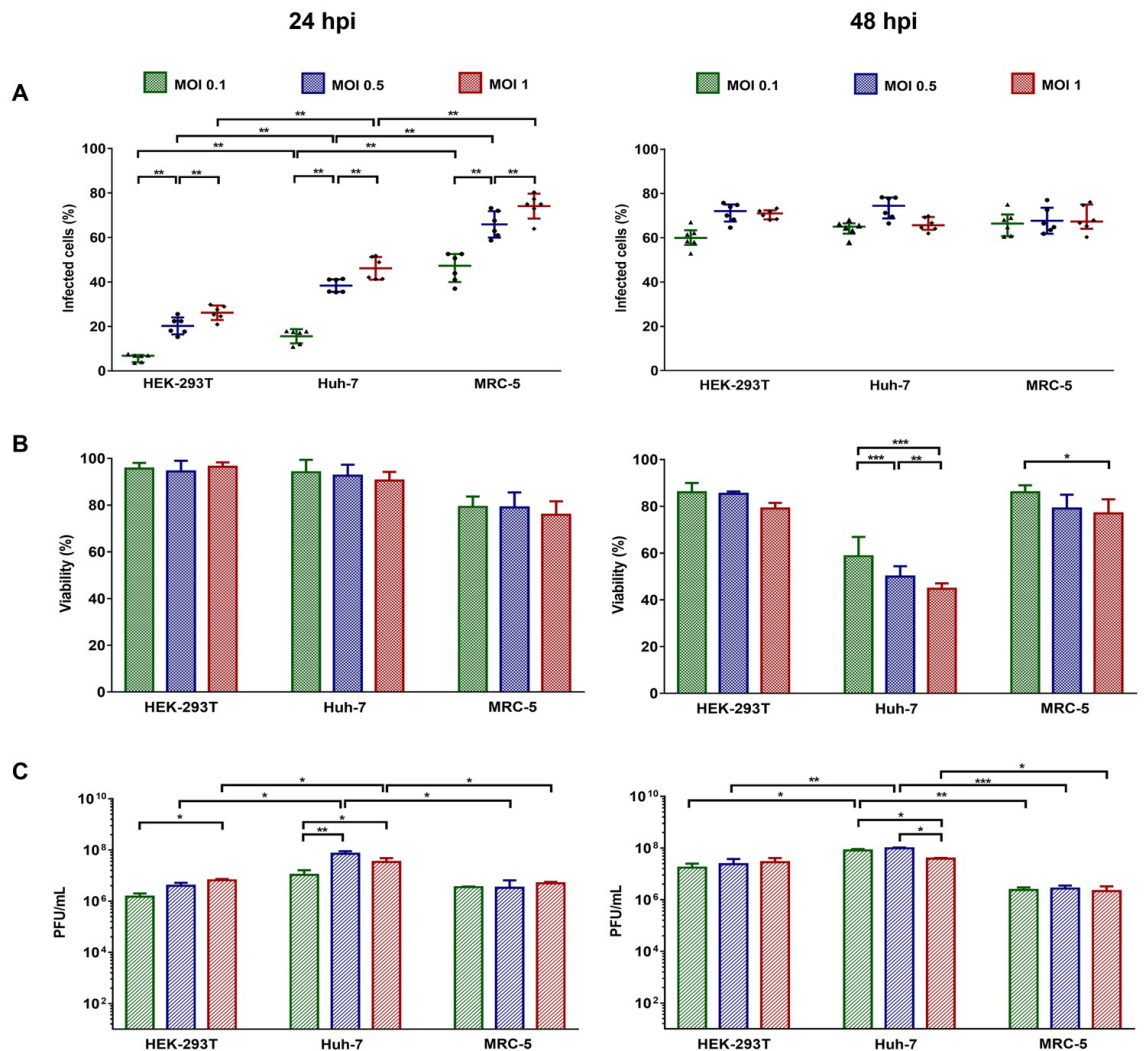
Evaluation of the infective and replicative capacity of the COL7624 CHIKV isolate. Cells were infected at the indicated MOIs. At 24 and 48 hpi, the cells were fixed, permeabilized, stained with anti-E1 CHIKV antibody, and analyzed by flow cytometry (A). Cell viability was analyzed using a resazurin assay (B). Levels of infectious virions in supernatants were measured by plaque assay on Vero cells and are expressed as the number of plaque-forming units (PFU)/mL (C). Significant differences between the groups were evaluated considering the non-parametric Mann–Whitney statistical test (*P < 0.05; **P < 0.01; and ***P < 0.001). Data are representative of three independent experiments (n = 3).

As the biological behavior of the Asian genotype has been poorly analyzed and the virus that was introduced and spread rapidly in the American continents belonged to a specific lineage that cause more severe disease than its ancestor, we evaluated the infective and replicative capacity of the COL7624 isolate in three human cell lines susceptible to infection. For this purpose, HEK 293T, Huh-7, and MRC-5 cells were infected at MOI values of 0.1, 0.5, and 1 for 24 and 48 h. Viral antigen detection was then performed by flow cytometry and immunofluorescence, cell viability was also assessed, and the viral particles released into the supernatant were quantified. For flow cytometry, cells were labeled with the antibody against the E1 structural protein, and the percentage of infected cells was determined (Fig 2). At 24 hpi, significant differences were observed between the cell lines at the three evaluated MOIs. At MOI 1, MRC-5, Huh7, and HEK293T cells reached infection percentages of 75%, 50%, and 30%, respectively. At 48 hpi, the three cell lines reached infection percentages close to 75% at MOI 1, and no significant differences were observed between the different MOIs analyzed (Fig 2A).

**Fig 3:**
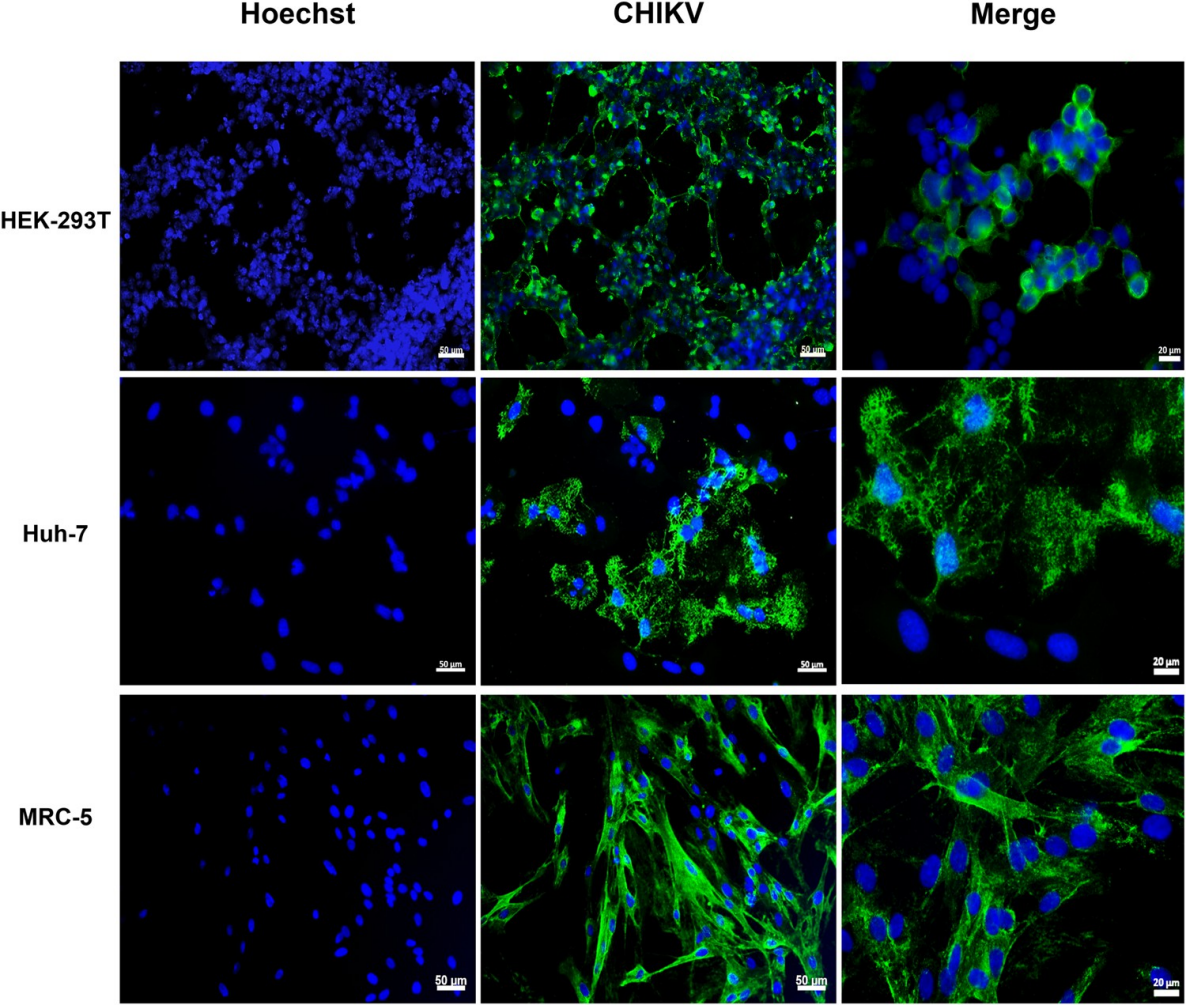
Assessment of CHIKV infection by immunofluorescence. Cells were exposed to CHIKV isolate at MOI 1. At 48 hpi, the cells were fixed, permeabilized, stained with anti-E1 CHIKV antibody.

A decrease in cell viability was observed after 24 h of infection; however, the cell viability remained above 90% in HEK293 and Huh-7 cells, while it fell below 80% in MRC-5 cells. At 48 hpi, Huh-7 cells were the most affected cells, reaching a viability loss of 50–60% at MOIs of 0.5 and 1, while the viability of HEK293T and fibroblasts remained close to 80% (Fig 2B). The impact of infection was also evident in immunofluorescence assays, where the loss in monolayer integrity and altered cell morphology were mainly observed in hepatocytes (Fig 3).

Assessment of the replicative capacity of the COL7624 isolate performed through the evaluation of infectious progeny present in the supernatant revealed no significant differences between the MOI values used for infection in MRC-5 cells. However, significant differences were observed in HEK293T and Huh-7 cells between the MOI values of 0.1 and 1 at 24 hpi, while there were no differences post 48 hpi (Fig 2C). Thus, using a higher viral inoculum did not significantly affect the viral replication, contrary to the effect on infection percentage and cell viability.

Evaluation of the differences between the cell lines revealed that Huh-7 cells released the highest number of infectious particles, reaching titers between 1 and 6 × 10^7^ PFU/mL, while MRC-5 cells reached titers in the order of 10^6^ PFU/mL, independent of the MOI or evaluation time (Fig 2C). Despite the low percentage of infection observed at 24 hpi (30%) for HEK293, the resultant infective viral progeny was similar to that of fibroblasts, in which the number of infected cells was higher (70%).

### Replication kinetics of the COL7624 isolate

**Fig 4:**
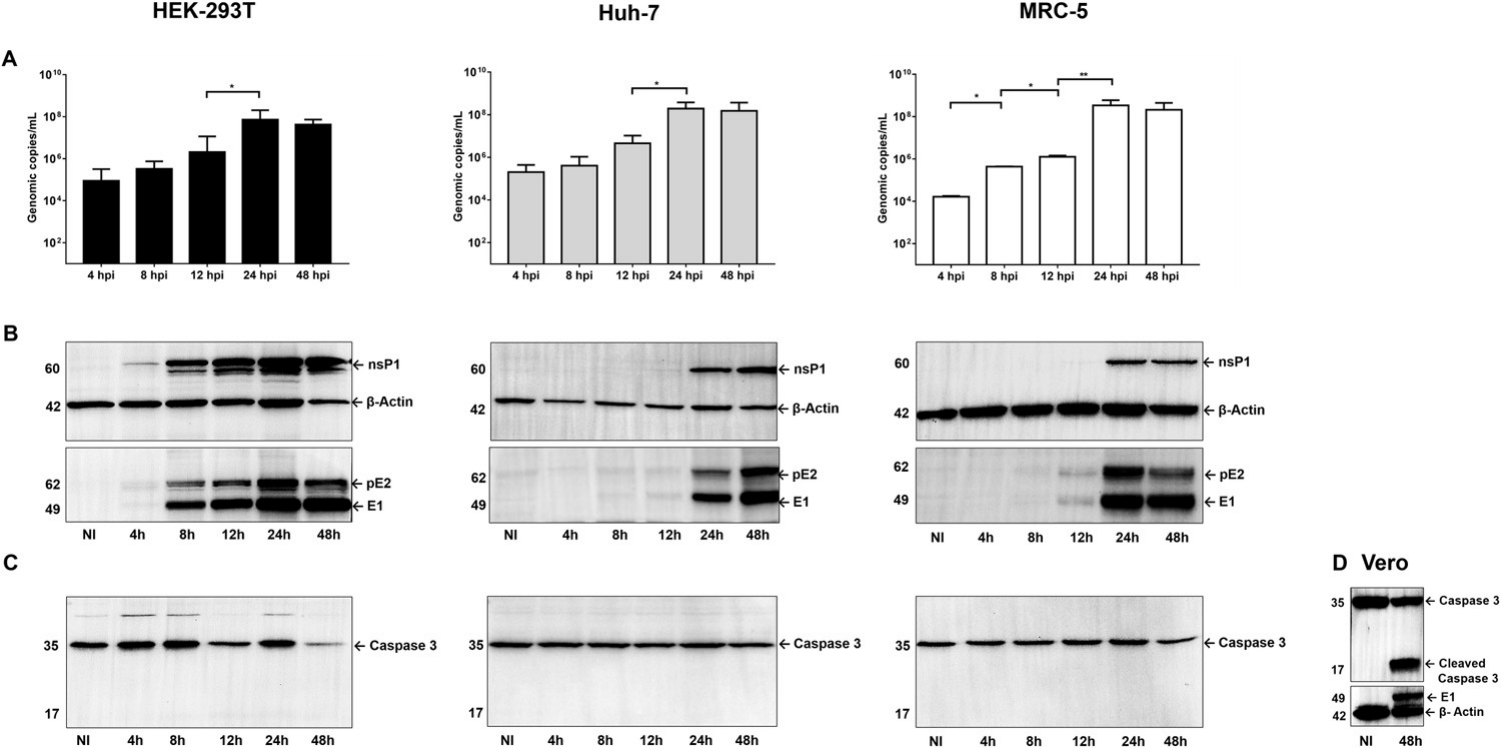
Kinetic analysis of the COL7624 isolate. Cells were infected at MOI 1 and harvested at the specified time points. Supernatants were collected, and the gRNA content was quantified by RT-qPCR assay based on E1 amplification (A). The cells were lysed and analyzed by western blotting using antibodies against nsP1 and E1/E2 proteins (B) and anti-caspase 3 (C, D). β- actin was used as a loading control. Statistical comparisons were performed using non-parametric Mann–Whitney tests (*P < 0.05; **P < 0.01; and ***P < 0.001).

The viral replication kinetics was evaluated in the three cell lines by infecting the cells at MOI 1 and collecting the supernatants as well as cells at 4, 8, 12, 24, and 48 hpi. The number of genomic copies in the supernatants was quantified by RT-qPCR, and the levels of viral proteins nsP1 and E1 in the cell lysates were determined by western blotting. Our results demonstrated viral particle release into the supernatant as early as 4 hpi, which increased until 24 h, with a significant increase between 12 and 24 h in the three cell lines. Interestingly, a slight decrease was observed at 48 hpi (Fig 4A). The detection of viral antigen presented some differences; nsP1, E1, and the 62 kDa E2 precursor were detectable from 8 hpi in HEK293T, while the same was detectable after 12 hpi in MRC-5 and Huh-7 cells. As for the genome, the levels of two viral proteins increased markedly between 12 and 24 h (Fig 4B). Given the effect on cell viability observed previously (Fig 2B), we evaluated if the observed effect was due to the activation of apoptosis. Accordingly, Caspase 3 activation by the proteolytic cleavage of procaspase was evaluated by western blotting. The detection of Caspase 3 as a single 35-kDa band and absence of the 17-kDa band in the cell lysates at the indicated time points demonstrated that the activation of programmed cell death did not occur or was not significant; consequently, the active caspase was not detectable (Fig 4C). However, in Vero cells, in which significant cytopathic effect and higher infection ratios were observed (S1 Fig), Caspase 3 activation was detected at 48 hpi (Fig 4D).

### Evaluation of immune responses against the CHIKV infection by the COL7624 isolate

**Fig 5:**
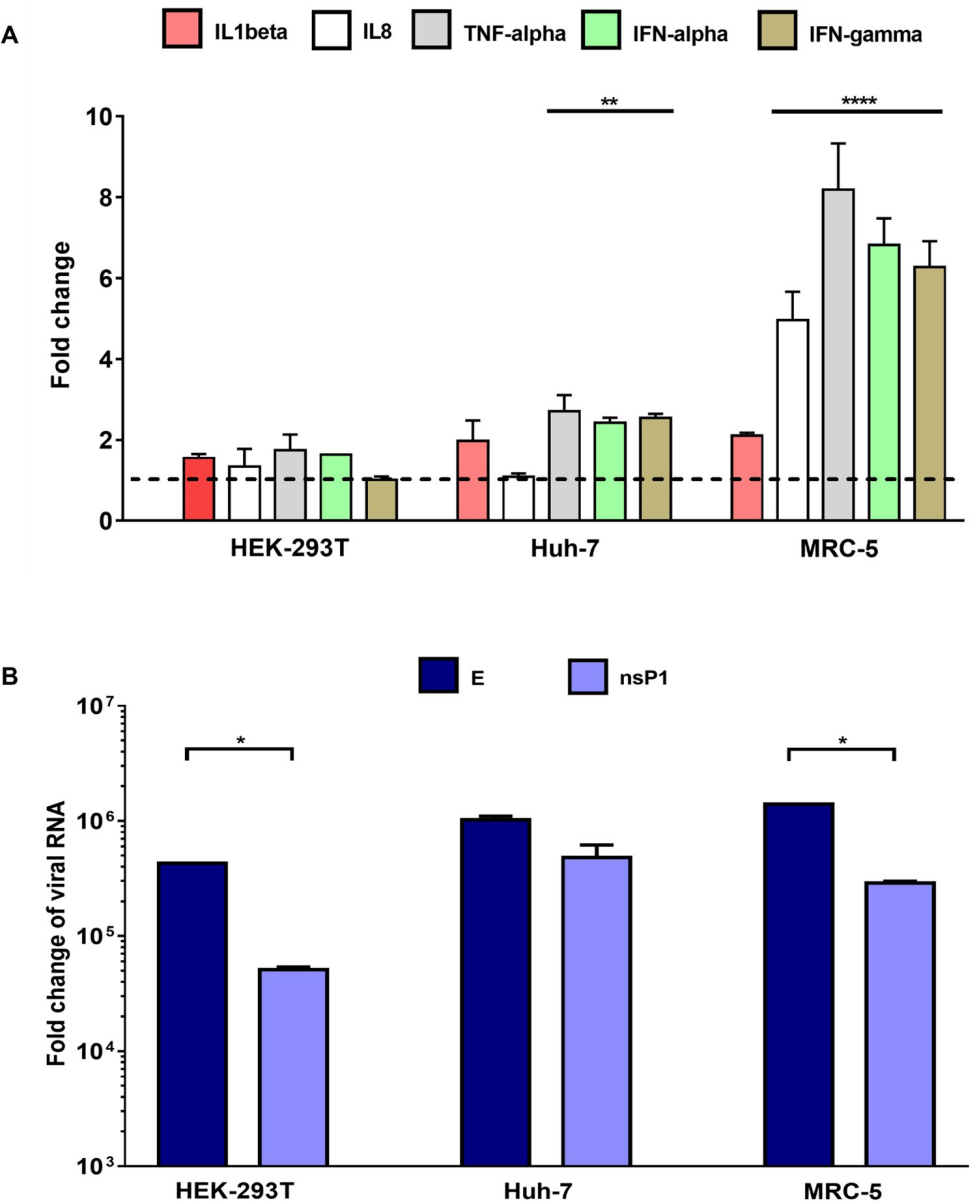
Gene expression levels of pro-inflammatory genes in CHIKV infected cells. Cells were infected at MOI 1 for 24 hpi and the RNA extracts were analyzed by RT-qPCR. Fold-change in mRNA levels of TNF-α, IFN-α, IL-1β, and IL-8 were calculated using mock-infected cells as control (A). Viral RNA levels (B). Data were analyzed with the One-way ANOVA test. Statistically significant differences are indicated: ** *P* < 0.01; *** *P* < 0.001; and **** *P* < 0.0001.

The transcription levels of tumor necrosis factor-α (TNF-α), interferon α (IFN-α), and interleukins (ILs) IL-1β and IL-8, which are involved in the pro-inflammatory response associated with the immuno-pathogenesis of CHIKV infection [37], were evaluated in cells infected at 24 hpi. Although the expression of these genes in all three assessed cell lines was higher than that in the mock-infected cells, their upregulation was evident in fibroblasts, while it was low in epithelial cells; as the fold change did not exceed 2 for any of the transcripts (Fig 5A). TNF-α was the most upregulated cytokine, with increases of 1.7, 2.7, and 8.1 folds in HEK293T, Huh-7, and MRC-5 cells, respectively, followed by IFN- α (1.6-, 2.5-, and 6.8-fold changes, respectively). The degree of activation of the immune response was consistent with the levels of viral RNA detected, with it being the highest in MRC-5 and the lowest in HEK293T cells. Furthermore, the expression of E1 was significantly greater than nsP1 in all the analyzed cell lines (Fig 5B).

## Discussion

From 2004 to 2011, the IOL lineage of the ECSA genotype was responsible for CHIKV fever outbreaks in East Africa, the Indian Ocean islands, India, Southeast Asia, and Europe, which spread rapidly and affected millions of people [8]. During 2005–2006, a large epidemic was documented in La Réunion island [1, 3], in 2006–2007, over one million cases were reported in India [8, 18] and through 2007–2009 the first cases of autochthonous transmission in Europe [19, 20]. Meanwhile, most outbreaks related to the Asian genotype were limited and geographically concentrated in Southeast Asia, China, and the Pacific islands, such as New Caledonia and Micronesia [4–7]. However, by the end of 2013, the Asian genotype reached the Caribbean and the Americas and promptly spread throughout the continents in a few months. This burst transmission was accompanied by an increase in atypical and severe manifestations of the disease [24, 25, 29–32]. Phylogenetic studies conducted with the complete genomic sequence of these viruses coincided with their grouping in the American lineage [7, 16, 22, 23]. Our analysis of the Asian genotype with sequences reported from 1953 to 2016 revealed numerous changes in the genome accumulated over the last 15 years, which led to a clear separation of the sequences into four clades whose members coincided geographically and temporally, in addition to detection of a close relationship between AAL and the virus that caused the 2012 Philippines outbreak (Fig 1, Table 2). Similar results were reported by Tan et al. in 2015; the viruses circulating in the Philippines and Indonesia from 1985 to 2014 were grouped into subclades denominated as B1, B2, and B3, and the sequences isolated in the Philippines in 2012 and the Caribbean in 2013 were classified under the subclade B3 [7].

According to our results, the Colombian isolate is a typical member of the AAL, with just three changes on the coding region as compared to other 46 American genomes, therefore, similar biological behavior *in vitro* could be expected for the other members. Our assays revealed that at 48 hpi, all the tested cell lines reached an infection proportion of over 65%, independent of the MOI used. However, as previously reported for other CHIKV genotypes [38–41], the COL7624 isolate also caused cell death as the infection progressed, being more deleterious at higher MOI values.

Previously, the isolate SZ1239 had shown good replicative capacity in five human cell lines, including HEK293T and HepG2 (hepatocyte carcinoma similar to Huh-7) wherein the infection at MOI 0.1 yielded 10^7^ to 10^8^ PFU/mL at 24 to 72 hpi [40]. Given that isolate SZ1239 clustered with other strains of the Asian genotype in the B3 subclade, which has been considered as a predecessor of the AAL lineage, we hypothesized that there are no apparent differences in the biological behavior between the Asian genotype causing CHIKV outbreaks in Indonesia and the Philippines (2010–2012) and the AAL, at least in mammalian cells under *in vitro* conditions [7, 40].

Similar findings were reported by Stapleford et al. (2016), wherein viral replication assays were performed with an infectious clone that was generated from a Caribbean sequence and an Asian genotype isolate from New Caledonia (NC2011) in Vero and BHK-21 cells showed both the viruses reached similar titers during the first 48 hpi. Strangely, the infectious clone (AAL) presented a replication 10 times higher than the Asian strain in C6/36 mosquito cells. These findings prompted them to suggest that the duplication at the 3’UTR end confers an adaptive advantage for replication in mosquito cells but does not affect replication in mammalian cells [22].

CHIKV replication occurs rapidly in susceptible cells, and infection induces apoptosis and an immune response involving the production of antiviral IFNs and pro-inflammatory cytokines [38, 39, 42–45]. Consequently, we evaluated the replication pattern of COL7624 CHIKV isolate, the activation of apoptosis pathways, and the expression of certain cytokines associated with activation of the immune response during the early phase of infection. Our results revealed the detection of viral particles at 4 hpi in the supernatants of all three cell lines, which progressively increased up to 24 hpi. Furthermore, the E1/E2 and nsP1 proteins reached detectable levels at 12 hpi and higher signal at 24 hpi, which coincides with the release of an elevated number of infectious particles into the cell culture supernatant, and no significant loss of cell viability. Remarkably, the Caspase-3 activation triggered by CHIKV infection was not observed in any of the tested cells even at 48 hpi, although we did not rule out a low percentage of apoptotic cells that could not be detected by western blotting.

Viral replication analyses conducted with the ECSA genotype have indicated earlier detection of viral proteins and a peak of viral progeny release after 12 hpi [42, 44, 45]. As replicative fitness depends on different variables, such as cell type, viral strain, and immune response activation; our results might not be comparable with those of previous reports. However, Teo et al. had reported a lower replicative capacity of a Caribbean isolate as compared to the one from La Réunion, as reflected in a lower percentage of infected cells, lower viral load, and minor viral titer at 12 hpi, but without significant differences at 24 hpi [42]. These findings are consistent with our observation of a later replication peak at 24 hpi for the COL7624 isolate.

Our results have demonstrated an increase in the transcription of IFN-α and TNF-α as well as a relationship between viral load and mRNA upregulation with immune response activation. Strikingly, the highest levels of intracellular viral RNA and upregulation in the five evaluated transcripts were observed in fibroblasts (Fig 5). The association between increased production of pro-inflammatory cytokines and the CHIKV viral load has been reported previously in both *in vitro* and *in vivo* studies and during the acute phase of infection in humans [36, 42, 46, 47]. IFNα is an essential mediator of the anti-CHIKV immune response [38], and its increased expression at 24 hpi potentially explains the decrease in viral replication observed at 48 hpi (Fig 2).

The over-expression of pro-inflammatory cytokines, including IL-1β, TNFα, IL8, and IFNs-α, that was observed in this study has been described in other *in vitro* cell models, such as the neuroblastoma line SHSY-5Y, primary human fibroblast-like synoviocytes (HFLS), monocyte-derived macrophages, and phorbol 12-myristate 13-acetate (PMA)-differentiated U937 macrophages [41, 47–49].

In conclusion, our study demonstrates that the Colombian isolate COL7624 is an Asian-American lineage member. It exhibited a high infective and replicative capacity, as indicated by the high production of infectious particles at 24 hpi. Infection with this isolate also induced an early and robust anti-inflammatory response in the cells, similar to the changes in the inflammatory mediators seen in cases with severe disease.

## Supporting information

S1 Fig Vero cells infection. Vero cells were exposed to CHIKV at MOI 0.5. At 48 hpi, the cells were fixed, permeabilized, stained with anti-E1 CHIKV antibody, and analyzed by flow cytometry. Dot plots based on CHIKV detection are shown and the results for three independent experiments. Upper panel: Bright-field image from the cell cultures.
(TIF)

S1 Raw images (PDF)
