# Supplementary figures and images for: Molecular and biological characterization of an Asian-American isolate of Chikungunya virus
Source: PLoS One. 2022 Apr 6;17(4):e0266450. doi: 10.1371/journal.pone.0266450 (PMC8985947; doi:10.1371/journal.pone.0266450)

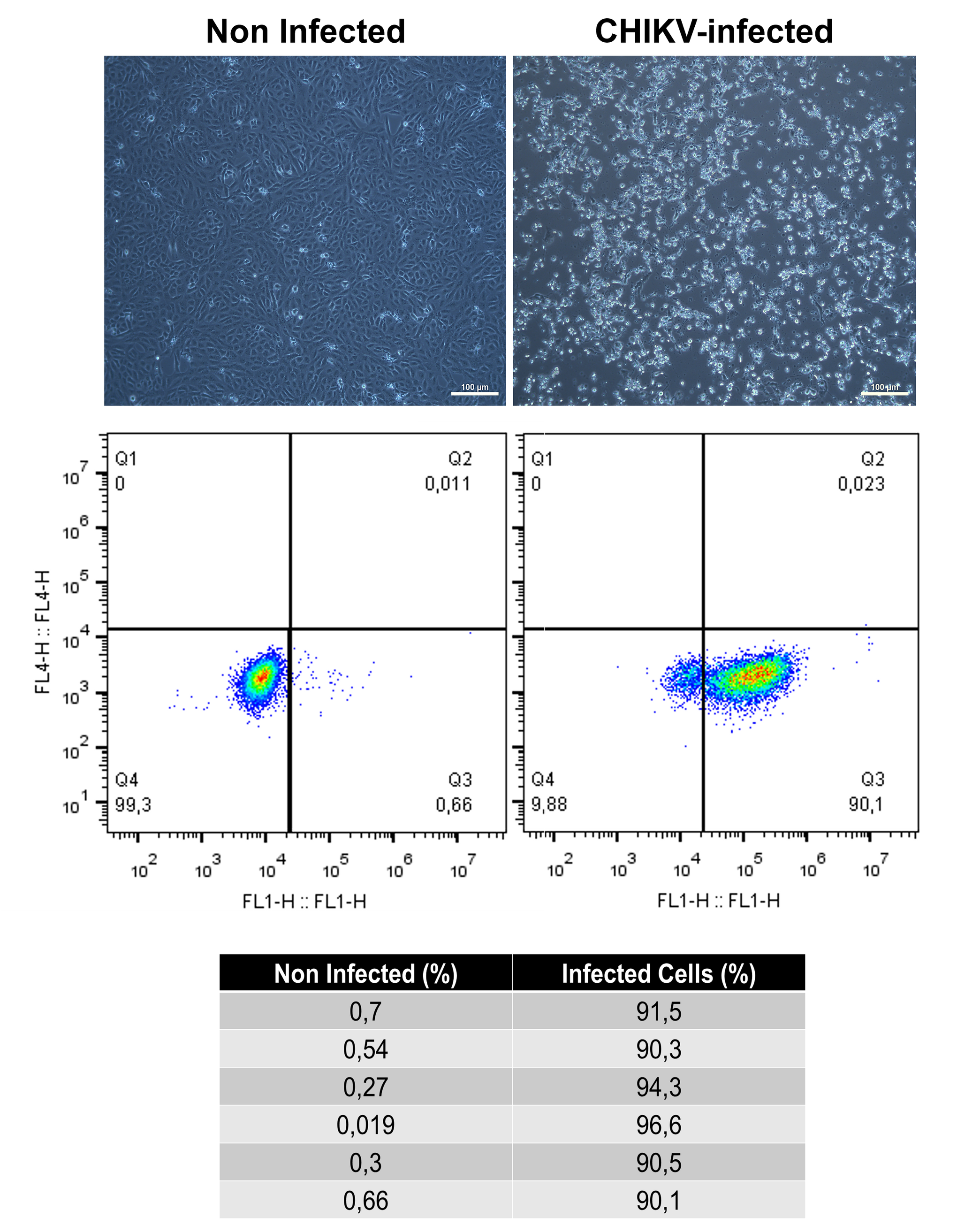

Supplement: S1 Fig — Vero cells were exposed to CHIKV at MOI 0.5. At 48 hpi, the cells were fixed, permeabilized, stained with anti-E1 CHIKV antibody, and analyzed by flow cytometry. Dot plots based on CHIKV detection are shown and the results for three independent experiments. Upper panel: Bright-field image from the cell cultures. (TIF) [file pone.0266450.s001.tif]
