# Supplementary material for: Molecular and biological characterization of an Asian-American isolate of Chikungunya virus
Source: PLoS One. 2022 Apr 6;17(4):e0266450. doi: 10.1371/journal.pone.0266450 (PMC8985947; doi:10.1371/journal.pone.0266450)

**Fig 4. Kinetic analysis.** Cells were infected at MOI 1 and analyzed at the indicated time points. Cell lysates were separated by SDS-PAGE, and the viral protein was assessed by western blot using antibodies against nsP1; E1/E2 proteins and anti-caspase 3. β- actin was used as a loading control. NI: non infected cells.

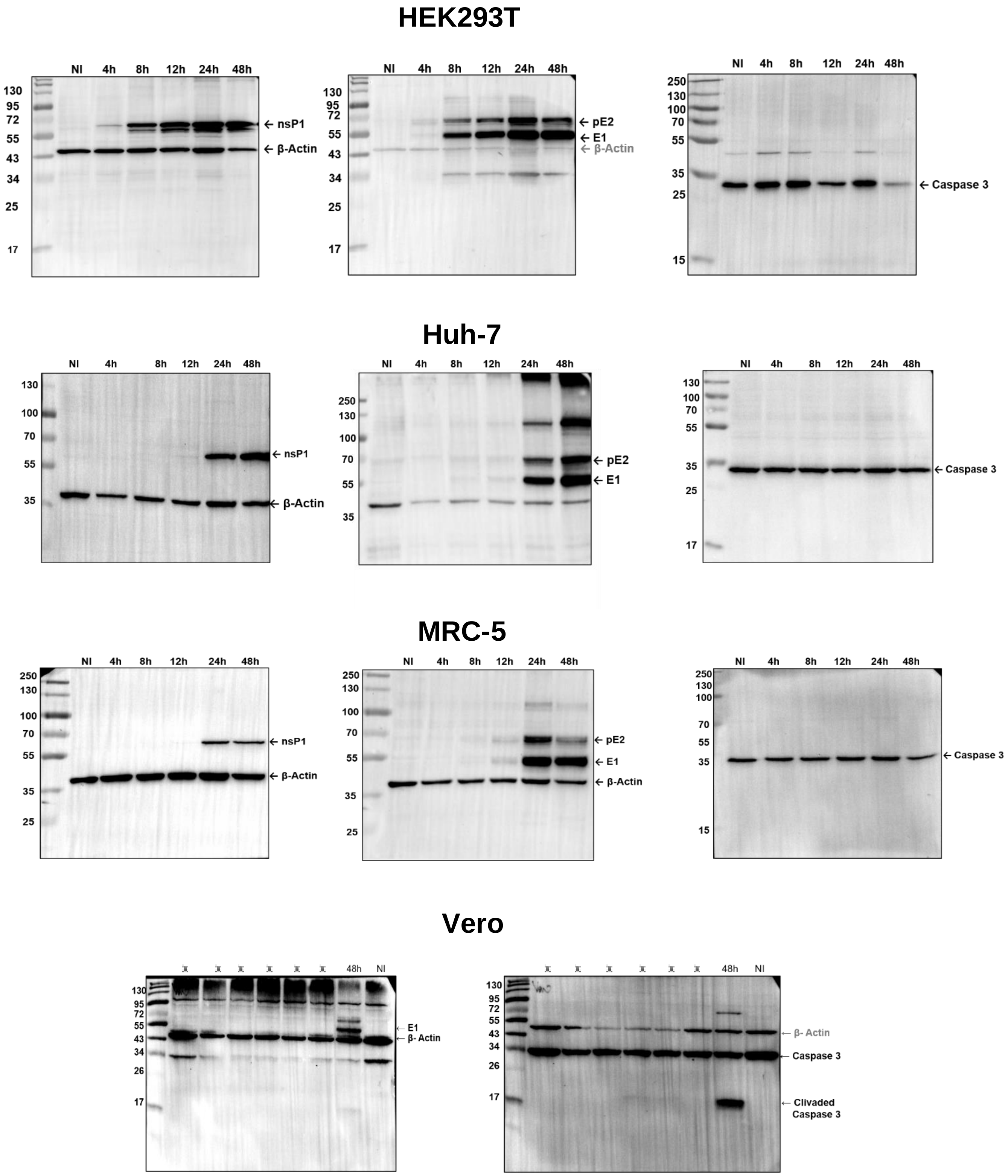

Supplement: S1 Raw images — (PDF) [file pone.0266450.s002.pdf]
